# Occurrence of HHIP gene CpG island methylation in gastric cancer

**DOI:** 10.3892/ol.2014.2518

**Published:** 2014-09-10

**Authors:** YU SONG, YUN ZUO

**Affiliations:** Department of Oncology, Zhangjiagang First People’s Hospital, Suzhou, Jiangsu 215600, P.R. China

**Keywords:** HHIP gene, gastric cancer, methylation

## Abstract

The present study aimed to observe the methylation status of the CpG islands at the human hedgehog interacting protein (HHIP) gene in gastric cancer tissues, peritumoral tissues and the AGS cell line, to analyze the association between the methylation status of the CpG islands and the tumorigenesis of gastric cancer. The HHIP mRNA expression in 60 human gastric carcinnoma tissues, peritumoral tissues and the gastric carcinoma AGS cell line were detected by reverse transcription polymerase chain reaction (RT-PCR). The HHIP methylation status of the promoter region in the gastric carcinnoma tissues and peritumoral tissues was detected by methylation-specific PCR (MSP). Prior to and following treatment with methyl transferase inhibitor 5-aza-2′-deoxycitydine (5-aza-dc), the HHIP mRNA expression level, the methylation status of the promoter region and the methylation site loci on the CpG islands in the AGS cells were detected by RT-PCR, MSP and bisulfite sequencing PCR (BSP), respectively. The correlation between the methylation status of the CpG islands at the HHIP promoter region and the HHIP mRNA expression level were analyzed. It was found that the expression level of the HHIP mRNA in the gastric carcinoma tissues was significantly lower than that in the adjacent tissues (0.82±0.38 vs. 1.60±0.26, respectively; P<0.001). No significant correlations were observed between the expression of HHIP mRNA and age, gender, tumor-node-metastasis stage, differentiation degree and presence of lymph node metastasis (P>0.05). The degree of methylation of the HHIP gene promotor in the peritumoral tissues (17.7±3.59%) was significantly lower than that in the gastric cancer tissues (62.9±6.14%) and in the AGS cells (99.7±0.67%) (P<0.05). Compared with prior to 5-aza-dc intervention, the HHIP mRNA expression level in the AGS cells was significantly increased subsequent to intervention (0.21±0.12 vs. 4.68±0.22; P<0.01), while the degree of methylation in the AGS cells was significantly decreased (90.2±0.67 vs. 10.1±0.21%; P<0.01), and the methylation sites in CpG islands were significantly reduced. The degree of HHIP methylation showed a negative correlation with the level of mRNA expression (r=−0.693; P<0.01). It can be hypothesized that a high degree of methylation of the HHIP gene promoter CpG islands in gastric cancer tissues and cells causes a decrease in HHIP mRNA expression, which may be involved in the carcinogenesis of gastric cancer.

## Introduction

Globally, gastric cancer is one of the most common cancers, the third most frequent malignancy and the second most common cause of cancer-related mortality annually (1,2). Gastric cancer is usually diagnosed in the later stages of the disease and has an extremely poor prognosis, as patients have unresectable, metastatic or recurrent gastric cancer with few available therapeutic options (3). Therefore, there is an urgent requirement to extensively investigate the molecular mechanisms of gastric carcinogenesis, and to develop novel therapeutic strategies for the control of gastric cancer.

Previous studies (4–6) have found that various methylation abnormalities can lead to gene inactivation and gene silencing, promoting the development of gastric cancer. The human hedgehog interacting protein (HHIP) gene is located on chromosome 4q31.21 31.3. HHIP was identified via the screening of a mouse cDNA expression library for proteins that bind to Shh. HHIP binds all three hedgehog (Hh) proteins with an affinity equal to that of Ptc-1 and thus, functions to negatively regulate the Hh pathway. Abnormalities in the activation of the Hh signaling pathway are one cause of the occurrence and development of tumors; the HHIP gene is a negative feedback factor of this pathway, which can directly inhibit the Hh pathway, and has been shown to have a significant role in development (7–9). At present, the expression of the HHIP gene in human gastric cancer and its association with the CpG island methylation status of the promoter has not been reported. The present study aimed to analyze the methylation of the HHIP gene in patients with gastric carcinoma.

## Materials and methods

### Clinical specimens

Surgical specimens from 60 patients with gastric cancer and adjacent normal tissues were collected from the Department of Surgery, Zhangjiagang First People’s Hospital (Jiangsu, China) between 2009 and 2013. All surgically resected tissue specimens were snap-frozen in liquid nitrogen until use. These specimens were examined by at least two experienced pathologists and tumor classification was made using the tumor-node-metastasis (TNM) classification. The patients consisted of 34 males and 26 females with an age range between 36 and 72 years (median, 60.82 years). According to the TNM staging system, 40 cases were stage II patients and 20 cases were stage III patients. A total of 32 tumors were well- and moderately-differentiated, while 28 were poorly-differentiated. A total of 24 tumors exhibited lymph node metastasis, whereas 36 cases were without lymph node metastasis. The study was approved by the ethics committee of Zhangjiagang First People’s Hospital. Written informed consent was provided by all patients.

### Cell culture and 5-aza-2′-deoxycytidine (5-aza-dc) treatment

The gastric cancer AGS cell line was purchased from the Shanghai Institute of Life Science Cell Information Center of the Chinese Academy of Sciences (Shanghai, China) and cultured in RPMI-1640 (Invitrogen, Carlsbad, CA, USA) supplemented with 10% fetal bovine serum (FBS), 100 μg/ml streptomycin and 100 U/ml penicillin at 37°C in a humidified atmosphere with 5% CO_2_. Cells in the logarithmic growth phase were cultured for ~24 h until they had reached 80% confluence, and then 5×10^6^ mol/l 5-Aza-dc (Sigma, St. Louis, MO, USA) was added. The liquid was changed once after 24h and then the cells were harvested following 72 h of continuous treatment.

### RNA isolation and reverse transcription polymerase chain reaction (RT-PCR)

Evaluate of HHIP mRNA expression using RT-PCR. Total RNA from the AGS gastric cancer cell line was isolated using TRIzol reagent (Shanghai Jingmei Bioengineering Co., Ltd., Shanghai, China) and converted into cDNA. The primer sequences of HHIP were as follows: forward, 5′-CTGCTTCTGTATTCAGGAGGTT-3′ and reverse, 5′-GGGATGGAATGCGAGGCTTA-3′, with an amplified fragment length of 229 bp. The primer sequences of the internal control, β-actin, were as follows: forward, 5′-AGAGCTACGAGCTGCCTGAC-3′ and reverse, 5′-AGCACTGTGTTGGCGTACAG-3′, with an amplified fragment length of 184 bp. The volume of the reaction agents was 20 μl, with reaction conditions of 95°C for 5 sec, 55°C for 5 sec and 72°C for 30 sec. The PCR products were subjected to 1.5% agarose gel electrophoresis analysis.

### Bisulfite conversion of DNA

Using phenol/chloroform, DNA was extracted, purified and transformed by the EZ DNA Methylation-Gold kit (Beijing Tianmo Technology Development Co., Ltd., Beijing, China).

### Methylation-specific PCR (MSP) for detection of HHIP gene methylation

The HHIP MSP primer was designed by ABI Methyl Primer Express v1.0 software (Applied Biosystems, Foster City, CA, USA). The methylation primer sequences of HHIP were as follows: forward, 5′-GTAGTAGTCGGGTAGTTTCGGAATTTTC-3′ and reverse, 5′-AAAAACGACTAACCGCGACG-3′, with an amplified fragment length of 190 bp. The non-methylation primer sequences were as follows: forward, 5′-AGTAGTTGGGTAGTTTTGGAATTTTTGG-3′ and reverse, 5′-AAAAACAACTAACCACAACA-3′, with an amplified fragment length of 188 bp. The volume of the reaction agents was 50 μl, with reaction conditions of 94°C for 30 sec, 60°C for 40 sec and 72°C for 50 sec. The PCR products were subjected to 1.5% agarose gel electrophoresis analysis.

### Bisulfite sequencing PCR (BSP) for detection of HHIP gene methylation sites

The HHIP BSP primer was designed by ABI Methyl Primer Express v1.0 software (Applied Biosystems). The primer sequences of HHIP were as follows: forward, 5′-GGGGAGGAGAGAGGAGTTTG-3′ and reverse, 5′-CCCCACCACCTCCCTACTAC-3′, with an amplified fragment length of 243 bp. The primer sequences of the internal control, β-actin, were as follows: forward, 5′-AGAGCTACGAGCTGCCTGAC-3′ and reverse, 5′-AGCACTGTGTTGGCGTACAG-3′, with an amplified fragment length of 184 bp. The volume of the reaction agents was 50 μl, with reaction conditions of 94°C for 30 sec, 60°C for 40 sec and 72°C for 50 sec. The BSP products were sent to Shanghai Shengong Biological Engineering Co., Ltd. (Shanghai, China) for sequence analysis.

### Statistical analysis

The data was analyzed using the χ^2^ test and Student t-test, and correlation analysis was performed using SPSS version 16.0 software (SPSS, Inc., Chicago, IL, USA). P<0.05 was considered to indicate a statistically significant difference.

## Results

### Expression of HHIP mRNA in human gastric cancer tissues

**Figure 1:**
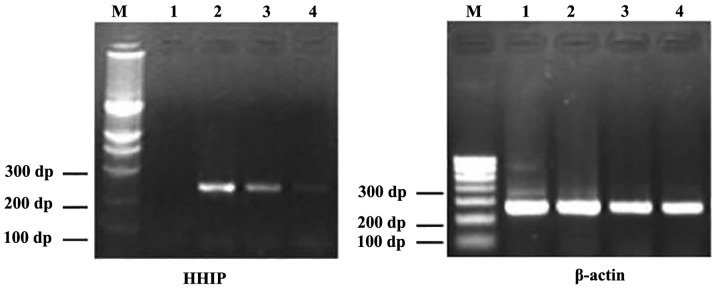
Expression of HHIP mRNA in gastric cancer, peritumoral tissues and gastric cancer AGS cells. M, marker; 1, AGS cells; 2 and 3, adjacent tissues; 4, gastric cancer tissues; HHIP, human hedgehog interacting protein.

Based on the RT-PCR results, HHIP mRNA was found to be expressed in the human gastric cancer tissues and adjacent gastric tissues, and was found to have almost no expression in the AGS cells (Fig. 1). The positive rate of HHIP mRNA expression in the gastric cancer tissues was 30% (18/60) compared with 66.67% in the adjacent normal tissues (40/60). The relative expression in the gastric cancer tissues was lower than that in the adjacent cancer tissues (0.8±0.38 vs. 1.6±0.26; P<0.001). No significant correlations were observed between the expression of HHIP mRNA and age, gender, TNM stage, differentiation degree and lymph node metastasis (P>0.05) (Table I).

### Detection of HHIP gene promoter methylation in gastric cancer tissues

**Figure 2:**
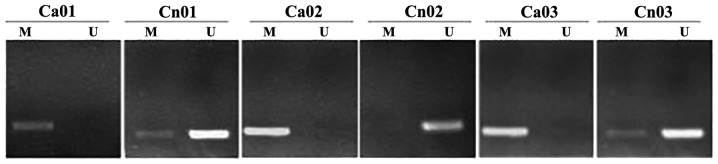
Detection of the methylation of human hedgehog interacting protein in gastric cancer tissues and adjacent tissues by methylation-specific polymerase chain reaction. Ca, gastric cancer tissues; Cn, peritumoral tissues; M, methylated; U, unmethylated.

Based on the results of the amplification procedure performed using MSP, a number of gastric cancer tissues were shown to exhibit methylated HHIP gene promoters compared with the adjacent normal tissues, as shown in Fig. 2. No significant difference in HHIP gene promoter region methylation was observed in the gastric cancer tissues and AGS cells (P>0.05), however, the HHIP gene promoter region methylation level was significantly lower in the adjacent normal tissues compared with the gastric cancer tissues and AGS cells (17.7±3.59 vs. 62.9±6.14 and 99.7±0.67%; all P<0.05).

### Effects of 5-aza-dc on the expression of HHIP mRNA and promoter methylation

**Figure 3:**
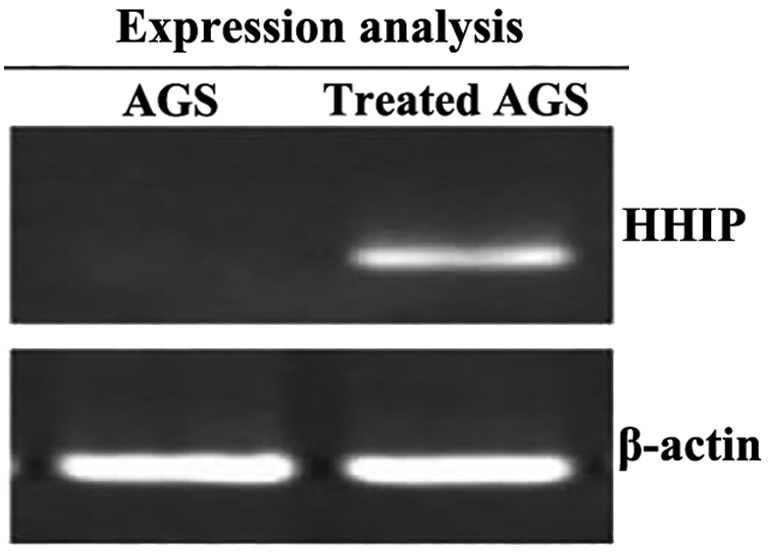
Expression of HHIP mRNA in gastric cancer AGS cells prior to and following 5-aza-2′-deoxycytidine treatment. HHIP, human hedgehog interacting protein.

**Figure 4:**
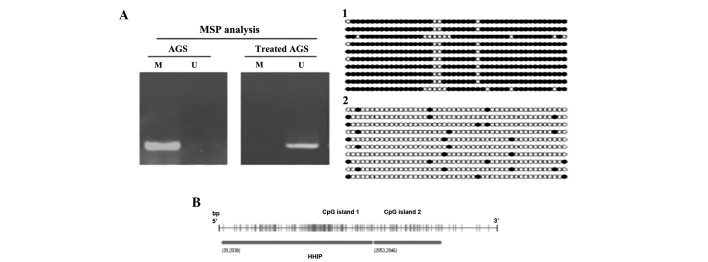
Differences in the methylation status of human hedgehog interacting protein in the gastric cancer AGS cell line prior to and following intervention with 5-aza-dc. 1, prior to 5-aza-dc treatment; 2, following 5-aza-dc treatment; U or ○, unmethylated; M or ●, methylated; 5-aza-dc, 5-aza-2′-deoxycytidine; MSP, methylation-specific polymerase chain reaction.(B) Software analysis of the promoter region locates two CpG islands in human hedgehog interacting protein (HHIP) mRNA.

Using RT-PCR, it was found that the AGS cells were activated following the intervention with 5-aza-dC; the expression of HHIP mRNA was significantly increased (0.21±0.12 vs. 4.68±0.22; P<0.01) (Fig. 3). Based on the results from the amplification procedure using MSP, the methylation level was shown to be significantly decreased following 5-aza-dC treatment (90.2±0.67 vs. 10.1±0.21%; P<0.01), as shown in Fig. 3. Spearman’s correlation analysis showed that HHIP gene promoter methylation was negatively correlated with mRNA expression (r=−0.693; P<0.001). With regard to the detection of the methylation status in the promoter region by the BSP method, the level of methylation significantly decreased following treatment, as shown in Fig. 4. Using CpG analysis (ABI Methyl Primer Express v1.0 software), the HHIP promoter region was determined to have 2 CpG islands; the first island is located from +39 bp to +2038 bp, the second island is located from +2053 to +2946 bp (Fig. 4B) The first island was used to design the HHIP primer sequence. It was found that the number of HHIP gene promoter CpG island methylation loci was significantly reduced following 5-aza-dC treatment.

## Discussion

Methylation of tumor suppressor genes has been found to cause numerous cancers and has been a focus of tumor research in recent years. DNA methylation is a form of chemical modification, which changes genetic expression without altering the DNA sequence. In a variety of human tumor genes, the DNA methylation level is low and a degree of regional hypermethylation coexists. Previous studies (10–12) have shown that 5′-CpG island hypermethylation leads to partial inactivation of tumor suppressor genes, which is a significant cause of the malignant transformation of cells. As a result treatment with demethylation drugs, the gene expresses a tumor suppressor function. It has been found that when using 5-azacytidine to act on promoter hypermethylation, the corresponding mRNA and protein expression can be restored in the tumor cells; this confirmed the fact that promoter methylation is the main cause of the inhibition of gene expression (13–15). One study has even proposed the establishment of a pattern of DNA methylation for multiple tumor-associated genes, to facilitate the diagnosis and differential diagnosis of early (16).

The HH signaling pathway is a vital signal transduction pathway that aids in regulating embryonic development. HHIP was first identified by screening a mouse cDNA expression library for proteins that bind to sonic hedgehog (Shh). HHIP binds all three Hh proteins with an affinity equal to that of Ptc-1, and functions to negatively regulate the Hh pathway. The expression of the HHIP gene, a negative regulator of Hh signaling, has been shown to be reduced in gastric cancer tissues, but retained in normal gastric tissues or atypical hyperplasia (17–19). The present study also found that the expression of HHIP mRNA in the gastric cancer tissues was significantly lower than that in the adjacent normal tissues (P<0.05), supporting the aforementioned results.

Accumulating evidence has shown that DNA methylation is closely associated with gastric cancer. Studies have found that hypermethylation exists in each stage of gastric cancer, even at the precancerous lesion stage. Lee *et al* (20) found that the p16 gene methylation level had positive correlation with gastric carcinogenesis. The level of p16 methylation may increase in intestinal metaplasioa, chronic gastritis, polypoid adenoma and adenocarcinoma. Berman *et al* (21) reported that, in 2003, 81% of digestive tract cancer cells, including esophageal, gastric, biliary and pancreatic cancer- derived cell lines, exhibit the expression of SHH and its receptor, PTCH. A study by Shahi *et al* (22) found that HHIP hypermethylated in pancreatic cancer cell lines. These data suggest that the Hh pathway is involved in the occurrence and development of gastric cancer. There is currently a lack of studies with regard to HHIP gene defects and mutations in gastric cancer. The number of studies analyzing HHIP gene methylation is even less.

This study analyzed the occurrence of HHIP gene CpG island methylation in gastric cancer. The study found that the level of HHIP gene promoter methylation in peritumoral tissues (17.7±3.59%) was significantly lower than that in gastric cancer tissues (62.9±6.14%) and AGS cells (99.7±0.67%) (P<0.05). The level of HHIP methylation increased significantly in normal gastric mucosa, gastric cancer and gastric cancer cell lines. Following intervention with 5-aza-dc, the methylation level of the AGS cell line decreased significantly and non-methylation of the HHIP promoter region was observed. The CpG methylation status was significantly reduced, whereas the HHIP gene was activated and the mRNA expression was significantly increased. Analysis showed that the mRNA expression level was negatively correlated with the methylation level. Therefore, HHIP gene CpG island hypermethylation may decrease the expression of HHIP, which maybe participate in the carcinogenesis of gastric cancer, therefore methylation in HHIP gene CpG islands may be a good detection index for gastric cancer.

In conclusion, HHIP gene promoter CpG island methylation may be associated with the carcinogenesis of gastric cancer, so the detection of the HHIP gene methylation level may be a novel clinical marker for the early diagnosis of gastric cancer. HHIP and the specific mechanism of gastric cancer require further study.

## Figures and Tables

**Table I:** Association between HHIP mRNA and the clinical features.

		HHIP		
		
Clinical features	n	Relative expression	t-value	P-value
Gender				
Male	34	1.3±0.02	0.545	0.596
Female	26	1.3±0.01		
Age, years				
<50	22	1.3±0.02	0.012	0.991
≥50	38	1.3±0.01		
TNM stage				
II	40	1.3±0.01	−0.031	0.976
III	20	1.3±0.01		
Differentiation				
Well and moderate	32	1.3±0.02	0.972	0.394
Poor	28	1.3±0.01		
Lymph node metastasis				
Yes	24	1.3±0.02	0.162	0.875
No	36	1.3±0.01		

HHIP, human hedgehog interacting protein; TNM, tumor-node-metastasis.
